# Link between Intestinal CD36 Ligand Binding and Satiety Induced by a High Protein Diet in Mice

**DOI:** 10.1371/journal.pone.0030686

**Published:** 2012-01-25

**Authors:** Danielle Naville, Adeline Duchampt, Michèle Vigier, Delphine Oursel, René Lessire, Hélène Poirier, Isabelle Niot, Martine Bégeot, Philippe Besnard, Gilles Mithieux

**Affiliations:** 1 Inserm, U855, Lyon, France; 2 University Lyon 1, Villeurbanne, France; 3 University of Lyon, Lyon, France; 4 CNRS, UMR 5200, Université Bordeaux Segalen, Bordeaux, France; 5 Physiologie de la Nutrition, AgroSup Dijon, UMR U866 Inserm/Université de Bourgogne, Dijon, France; Cornell University, United States of America

## Abstract

CD36 is a ubiquitous membrane glycoprotein that binds long-chain fatty acids. The presence of a functional CD36 is required for the induction of satiety by a lipid load and its role as a lipid receptor driving cellular signal has recently been demonstrated. Our project aimed to further explore the role of intestinal CD36 in the regulation of food intake. Duodenal infusions of vehicle or sulfo-N-succinimidyl-oleate (SSO) was performed prior to acute infusions of saline or Intralipid (IL) in mice. Infusion of minute quantities of IL induced a decrease in food intake (FI) compared to saline. Infusion of SSO had the same effect but no additive inhibitory effect was observed in presence of IL. No IL- or SSO-mediated satiety occurred in CD36-null mice. To determine whether the CD36-mediated hypophagic effect of lipids was maintained in animals fed a satietogen diet, mice were subjected to a High-Protein diet (HPD). Concomitantly with the satiety effect, a rise in intestinal CD36 gene expression was observed. No satiety effect occurred in CD36-null mice. HPD-fed WT mice showed a diminished FI compared to control mice, after saline duodenal infusion. But there was no further decrease after lipid infusion. The lipid-induced decrease in FI observed on control mice was accompanied by a rise in jejunal oleylethanolamide (OEA). Its level was higher in HPD-fed mice than in controls after saline infusion and was not changed by lipids. Overall, we demonstrate that lipid binding to intestinal CD36 is sufficient to produce a satiety effect. Moreover, it could participate in the satiety effect induced by HPD. Intestine can modulate FI by several mechanisms including an increase in OEA production and CD36 gene expression. Furthermore, intestine of mice adapted to HPD have a diminished capacity to modulate their food intake in response to dietary lipids.

## Introduction

CD36 is a multifunctional plasma membrane glycoprotein expressed by a broad variety of tissues [1], [2]. This receptor-like protein is able to bind multiple compounds such as oxidized LDL [3], collagen [4] or thrombospondin [5]. It also displays a very high affinity (i.e. in nanomolar range) for long-chain fatty acids (LCFA). Despite its multifunctionality, CD36 generally plays a specific role in a given cell type. For example, it is involved in collagen-mediated cytoadhesion in platelets, whereas it mediates LCFA uptake in myocytes. This cell specificity of function probably results from both cellular context (genotype and microenvironment) and aspects of CD36 itself. Indeed, regulation of the gene encoding this receptor is unusually complex [6] and this protein is subject to several post-translational modifications in a tissue-specific manner [7]. Recently, CD36 has been identified in rodent gustatory papillae as a lipid sensor involved in both the preference for fatty foods and cephalic phase of the digestion [8], [9]. CD36 is also found in the small intestine, in which its mRNA and protein levels are higher in the proximal parts (duodenum and jejunum), the main sites of fat absorption [10], [11]. Moreover, its localization is strictly restricted to the brush border membrane of differentiated enterocytes [10], [12], [13]. Although this location raises the possibility of an involvement in lipid uptake by intestinal cells, the specific role of CD36 in the small intestine is not yet fully understood. If comparison between wild-type and CD36-null mice has clearly shown an implication of CD36 in the formation, secretion and clearance of intestinal lipoproteins [14], [15], its involvement in FA uptake remains more elusive [16]. Indeed, some investigators report that there is no alteration of global LCFA absorption in *Cd36*-invalidated mice [13], [16], while others suggest that CD36 plays a role in LCFA uptake in the duodenum and proximal jejunum, but not the distal intestine [17]. Alternatively, it has been demonstrated that intestinal CD36 exerts also a role of lipid receptor, driving cellular signals stimulating chylomicron synthesis [13].

It is known that a lipid infusion into the intestinal lumen leads to a decrease in food intake [18] through mechanism which remains to be clarified. It was recently shown that this phenomenon could be in part related to the generation/mobilization of the lipid messenger oleoylethanolamide (OEA) by the jejunum mucosa, specifically in response to dietary lipids [19]. Using mice displaying a total invalidation of the *Cd36* gene, Schwartz *et al* have reported that hypophagic effect of dietary fat requires the presence of CD36, because CD36-null mice are insensitive to the feeding inhibitory effects of duodenal lipid infusion [19]. They conclude that intestinal CD36, by facilitating oleate uptake as a precursor of OEA and/or its synthesis could raise the production of OEA. According to our recent data [13], an involvement of CD36 as lipid receptor at the origin of cell signalling, is an alternative hypothesis explaining its effect on OEA quantity in jejuna mucosa [19].

To further explore the role of intestinal CD36 in the regulation of feeding behaviour, impact of an acute duodenal infusion of nutritional (lipids) and/or pharmacological (sulfo-N-succinimidyl oleate, SSO) CD36 ligands on the regulation of food intake was studied in wild-type and CD36-null mice. High-protein diets (HPD) are known to induce a prolonged decrease in food intake [20], [21]. To assess the relative anorexic effect of dietary lipids, we next explored whether the hypophagic effect of lipids was identical in mice subjected to a standard (control) or a HP diet.

## Materials and Methods

### Animals

All procedures were performed with the approval of the Regional Committee of Ethics for Animal Experiments.

Wild-type (Harlan, Le Marcoulet, France) and CD36 null male C57Bl/6J [22] mice were housed in a ventilated cabinet at 21°C with inverted light/dark cycle (darkness from 1:30 pm to 1:30 am) and free access to water and standard chow (A04, SAFE, Augy, France). For the intraduodenal infusion experiments, 5-week old mice were fasted for 6 hours and anesthetized using isoflurane throughout the surgical procedure. A silicone catheter (0.31 mm, inner diameter) was placed in the duodenum through a small incision and fastened using surgical adhesive (3M Vetbond tissue adhesive, Centravet, Lapalisse, France). The catheter was threaded under the skin to exit through an incision in the back of the neck. Duodenal catheters were flushed every day with a mixture of polyvinylpyrrolidone and marbocyl to avoid clogging of the catheter, mixture supplemented every two days with ketoprofen. Mice were allowed to recover for one week after surgery. Catheterization allowed us to infuse solutions directly into the duodenum of awake mice, after a 5-hour daytime fast.

### Duodenal infusions

#### Experiment 1: Effect of a lipid infusion on short-term food intake

**Figure 1:**
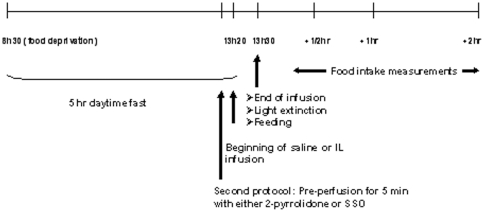
Experimental protocol and infusions.

After surgery, control mice were maintained on standard chow and infused, in a first series of experiments, with either isotonic saline solution or 20% Intralipid emulsion (IL from Fresenius Kabi France, Sèvres. It contains 20% soybean oil, 1.2% egg yolk phospholipids and 2.25% glycerine in water), at a rate of 6 µL/min during 10 min (0.12 Kcal infused). The end of the infusion/refeeding corresponds to the beginning of the dark period when mice were disconnected from the infusion pump. Food intake (one pellet of standard chow was weighed immediately before and after the test) was measured 30 min, 1 and 2 hours after the end of the infusion (Fig 1). Each animal was tested for each treatment condition alternatively, which means that each animal was its own control. Following feeding tests, mice were killed by cervical dislocation 45 min after the end of one type of experimental perfusion and different parts of the small intestine as well as total hypothalamus were recovered.

#### Experiment 2: Effect of sulfo-N-succinimidyl oleate on food intake measured after saline or Intralipid perfusion

In a second series of experiments, control and CD36-null mice (kind gift of Dr. Maria Febbraio from Lerner Research Institute, Cleveland, OH, USA) were infused prior to the saline and IL infusions, with either 2-pyrrolidone, Pyrr (vehicle) or sulfo-N-succinimidyl oleate, SSO (20 µM solution infused at a rate of 3 µL/min during 5 min). To avoid the infusion of dimethylsulfoxide, DMSO (the classical solvent of SSO), we used 2-pyrrolidone which has the advantage to make SSO soluble (F. Falson, personal communication) and to be miscible in water. The same mouse was alternatively exposed to the different infusions: pre-infusion of either 2-pyrrolidone or SSO followed by either saline or IL infusion (Fig 1). SSO specifically binds to CD36 and arrests the LCFA transport into adipocytes [1]. It was used in these experiments to specifically inhibit CD36 present in intestine cells. Food intake was measured as above.

#### Experiment 3: High-protein diet and lipid sensing

Five-week old male wild-type and CD36 null male C57Bl/6J mice were maintained either on standard chow or on an isocaloric protein-enriched diet, HPD (SAFE, 54% protein *versus* 16.1% in standard chow) up to 30 days (Table 1). Weight and food intake were recorded every day. Food intake was expressed as grams of food consumed by gram of body weight to obviate any differences in body weight between mice. Three groups of wild-type HPD mice were separated and maintained on HP diet for 4, 12 and 30 days. At the end of the experiments, mice were killed by cervical dislocation after a 6h-fast. Blood and different parts of the small intestine were recovered either as total tissue (RNA preparation) or the mucosa was scraped off with a spatula (proteins), frozen immediately in liquid nitrogen and stored at −80°C until used.

**Table 1:** Composition of the different diets.

% mass	Standard diet	HP diet (casein/Soya)
**lipids**	3.1	5
**carbohydrates**	60	10
**proteins**	16.1	54
	2.9 kcal/g	2.99 kcal/g

For infusion experiments, surgery was performed as described above on control or 12 day-HPD fed mice of same age. NaCl and IL perfusions were performed as above, each animal being its own control. HPD mice were fed the same HPD food before and after the infusion experiments. As above, mice were divided into different experimental groups for analyses of gene expression alteration elicited by one type of experimental infusion. Duodenum and the proximal part of the jejunum were recovered 45 min after the end of the infusion and the mucosa was scraped off with a spatula (OEA measurement), frozen immediately in liquid nitrogen and stored at −80°C until used.

### Plasma and tissue measurements

Commercially available EIA kits were used to measure the plasma level of insulin (Crystalchem, Chicago, USA) and Cholecystokinin, CCK (Phoenix Europe, Karlsruhe, Germany). Plasma glucose and triglycerides were measured using commercial kits (BioMérieux, Marcy-l'Etoile, France).

For lipid analysis, lipids were extracted by 2mL of CHCL3/MeOH (2/1,v/v) at 85°C for 1h. After addition of 2mL NaCl (2.5% w/v), the extract was vortexed and the above phase discarded. The chlorofomic phase was evaporated and dried under a nitrogen stream and the lipids were dissolved in 100 µL of CHCL3/MeOH (2/1,v/v). The lipids were separated by TLC using chloroform/methanol/1-propanol/methyl acetate/0.25% KCl (10/4/10/10/3.6, by vol.) or pyridine/chloroform/formic acid (25/15/3.5, by vol.). The N-oleoyl-phosphatidylethanolamine (NOPE) is identified by comparison of the Rf with the N-acylphosphatidylethanolamine (NAPE) used as a standard. The spots corresponding to the NOPE were scraped and subjected to a transesterification for 1 h at 80°C in the presence of 3 mL 5% sulfuric methanol containing C17:0 fatty acid as internal standard. The fatty acids were separated by GC and the lipids were quantified by comparison of their peak areas with the standard C17:0 as described by Testet et al [23].

### Quantitative RT-PCR (RT-qPCR)

Quantitative RT-PCR (RT-qPCR) was used to study the expression of different target genes. Total RNA was extracted from tissue samples using TRIzol (Invitrogen, Cergy-Pontoise, France) according to the manufacturer's protocol. All samples were treated by DNase I (Invitrogen) before the reverse transcription. First strand cDNAs were prepared using one µg RNA and the Moloney Murine Leukemia Virus Reverse Transcriptase, MMLV (Fermentas, St. Rémy-Les-Chevreuse France) in the presence of oligo(dT) primers (Fermentas). The qPCR reactions were performed using the Light Cycler Fast Start DNA Master SyBR Green I kit (Roche, Meylan, France) in the presence of specific primer pairs (Table 2). Samples (in duplicate) were quantified by comparison with a standard curve obtained by dilutions of purified target cDNA fragment and mRNA level was expressed as a ratio between values obtained for the target gene versus values obtained using the housekeeping genes L19 (intestine) and GAPDH (hypothalamus).

**Table 2:** Primers used for the qPCR (from Eurogentec, Seraing, Belgique).

genes		sequences: 5′->3′
CD36	sense	gatgacgtggcaaagaacag
	antisense	tcctcggggtcctgagttat
L19	sense	agattgaccgtcatatgttgtatca
	antisense	tttcgtgcttccttggtcttaga
POMC	sense	atgccgagattctgctacagtcg
	antisense	ttcatctccgttgccaggaaacac
AGRP	sense	ctcaagaagacaactgcaggac
	antisense	tgaagaagcggcagtagcac
GAPDH	sense	ttccagtatgactccactcacg
	antisense	agactccacgacatactcagca

### Western Blotting

The jejunum was cut in three parts of equal size and mucosa was scraped off from the first part (proximal jejunum). Ten µg of proteins, prepared from jejunal mucosa, were separated by SDS-PAGE 10% and transferred to a PVDF membrane (Immobilon-P transfer membrane, Millipore, Saint-Quentin-en-Yvelines, France). Immunoblotting was performed using goat antibodies directed against mouse CD36 (1,000-fold dilution, R&D Systems Europe, Lille, France) or mouse monoclonal antibodies directed against mouse beta-actin (1,000-fold dilution; Sigma-Aldrich, Saint Quentin Fallavier, France). Goat anti-mouse (BioRad Laboratories, Marnes-le-Coquette, France) and donkey anti-goat IgG (Santa Cruz Biotechnology, Tebu-bio France, Le Perray en Yvelines) were used as secondary antibodies for beta-actin and CD36, respectively. Blots were revealed using the Immobilon Western Blotting Chemiluminescent HRP Substrate (Millipore SAS, Molsheim, France). Quantitation of the bands was performed using Adobe Photoshop software.

### Statistics

All statistical analyses were performed using one-way ANOVA, followed by post hoc testing with Fisher's protected least square difference test (PLSD), with the aid of Statview 5.0 software package (SAS Institute Inc. Cary, NC 27513). Results were expressed as mean ± SEM and differences were considered significant at P<0.05.

## Results

### Binding of intestinal CD36 with the pharmacological drug SSO reproduces the lipid-mediated inhibition of food intake in refed mice

**Figure 2:**
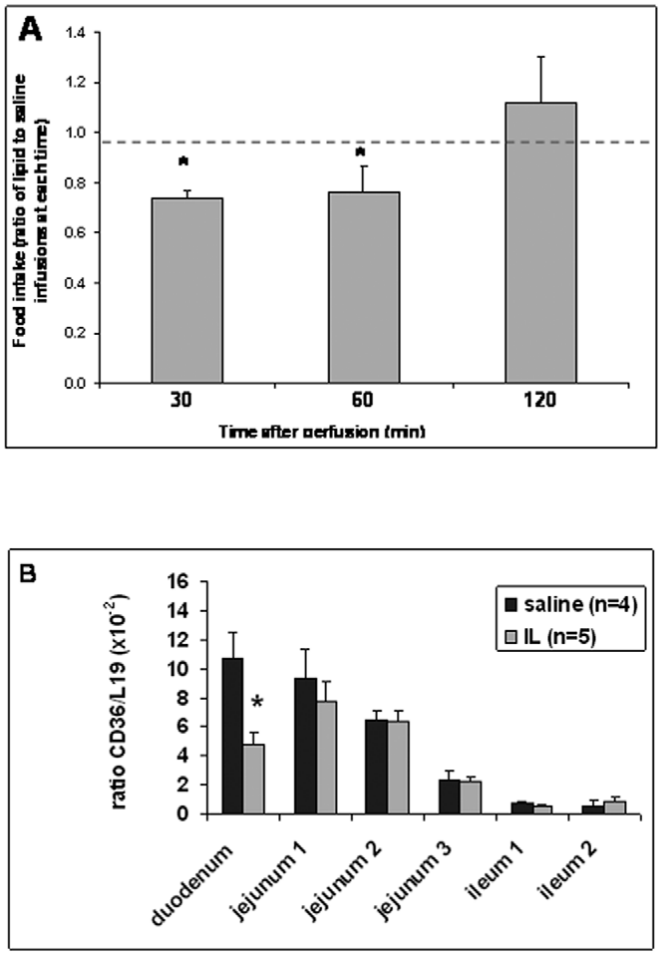
Food intake and CD36-encoding mRNA level in segments of the small intestine after intra-duodenal infusions. (**A**): Food intakes were measured 30 min, 1hr and 2 hr after the end of the infusion and were not cumulated. Each type of infusion was performed several times on the same animal. * P<0.05 relatively to saline infusion (for each period of time). The dotted line corresponds to the reference value at each time (mice infused with saline). The number of experiments (n = 16) corresponds to different animals. (**B**): The expression of the gene encoding CD36 was measured by RT-qPCR on RNA samples obtained 45 min after the end of the infusion. Results were expressed as mean ± SEM (CD36 *versus* L19). * P<0.05 differences between saline and IL infusions for each part of the small intestine.

To determine the kinetic of duodenal lipid infusion on food intake, fasted mice bearing a permanent duodenal catheter, were infused with saline or 20% IL (6 µl/min for 10 min). Food intake was significantly lower 30 and 60 min after the end of luminal infusion of IL as compared to saline solution. For example, food intake dropped from 0.33±0.02g (0.96kcal) to 0.24±0.01g (0.70 kcal) in control and IL mice 30 min after infusions, respectively (Fig 2-A). This satietogen effect was transient since it was not retrieved 120 min after the end of infusion (Fig 2-A).

Expression of CD36 encoding gene was measured by RT-qPCR in intestine serially divided in 6 segments of equal size, according to previously described procedures [24]. Consistent with previous published data [10], [11], a progressive decrease in CD36 mRNA levels was found from duodenum to ileum in control mice (Fig 2-B). In contrast, a 2-fold decrease in CD36 gene expression was found in duodenum of IL-infused mice as compared to controls (Fig 2-B). There was no difference between saline and IL infusions in the other parts of the small intestine suggesting that the amount of lipids infused in duodenum was too small to reach another part of the intestine and affect the expression of *Cd36* in jejunum and ileum (Fig 2-B). No significant differences in Agouti related Protein (AgRP) and Proopiomelanocortin (POMC) gene expression between saline and IL-infused mice were found in hypothalamus removed 45 min after the end of infusions (data not shown). Thus, the decrease in food intake mediated by an acute infusion of minute quantities of IL in duodenal lumen was not due to any alteration in the expression of the genes that are directly involved in the control of food intake at the hypothalamic level.

**Figure 3:**
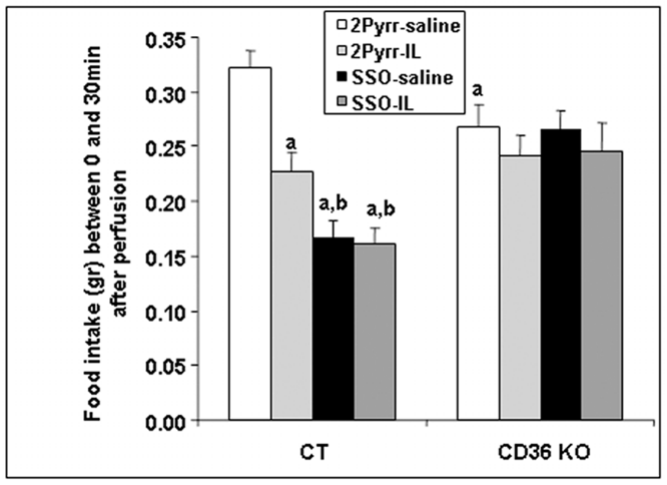
Food intake after different intra-duodenal infusions in wild-type and CD36-invalidated mice. Food intakes were measured 30 min after the end of each type of infusion performed either on wild-type (CT, n = 15) or CD36-invalidated mice (n = 9). **a**: P<0.05 relatively to Pyrr/saline infusion in CT; **b**: P<0.05 relatively to Pyrr/IL infusion in CT.

To further explore the intestinal CD36 implication either as a AGLC transporter [19] or a putative receptor [13] in the lipid-mediated control of food intake, pharmacological manipulation of the ligand binding site of CD36 was performed using an infusion of the sulfosuccinimidyl-oleate (SSO) into the duodenal lumen prior to IL or saline treatment in wild-type and CD36-null mice. SSO is known to be a specific and irreversible ligand for CD36. By this reason, it is likely that SSO is able to bind to intestinal CD36 receptor as well as inhibit lipid binding. As expected, a decrease of food intake took place 30 min after infusion of IL in wild-type mice (Fig 3). Surprisingly, a more drastic effect was found in SSO-treated wild-type mice in which subsequent infusion of IL did not induce an additive inhibitory effect on food intake (Fig 3). Thus, the irreversible binding of SSO to intestinal CD36 was sufficient to reproduce the satietogen effect of IL infusion. To verify this interpretation, we performed the same protocol in CD36-null mice. Basal food intake measured 30 min after saline infusion was significantly lower in CD36^-/-^ mice than in WT (Fig 3). When CD36 gene is lacking, neither SSO alone nor IL infusion alone or in association with SSO induced any change in food intake (Fig 3). Therefore, the binding of intestinal CD36 with a pharmacological ligand is sufficient to induce satiety. It is noteworthy that this effect is not secondary to lipid-mediated changes in the satiety hormone CCK since its plasma levels remained similar between the different groups (data not shown).

Taken together, these data are consistent with a role of CD36 in satiety as lipid receptor able to detect the presence of dietary lipids in intestinal lumen rather than lipid transporter as previously suggested [19].

### Chronic high protein diet affects intestinal CD36 gene expression, lipid-induced OEA synthesis and satiety

**Figure 4:**
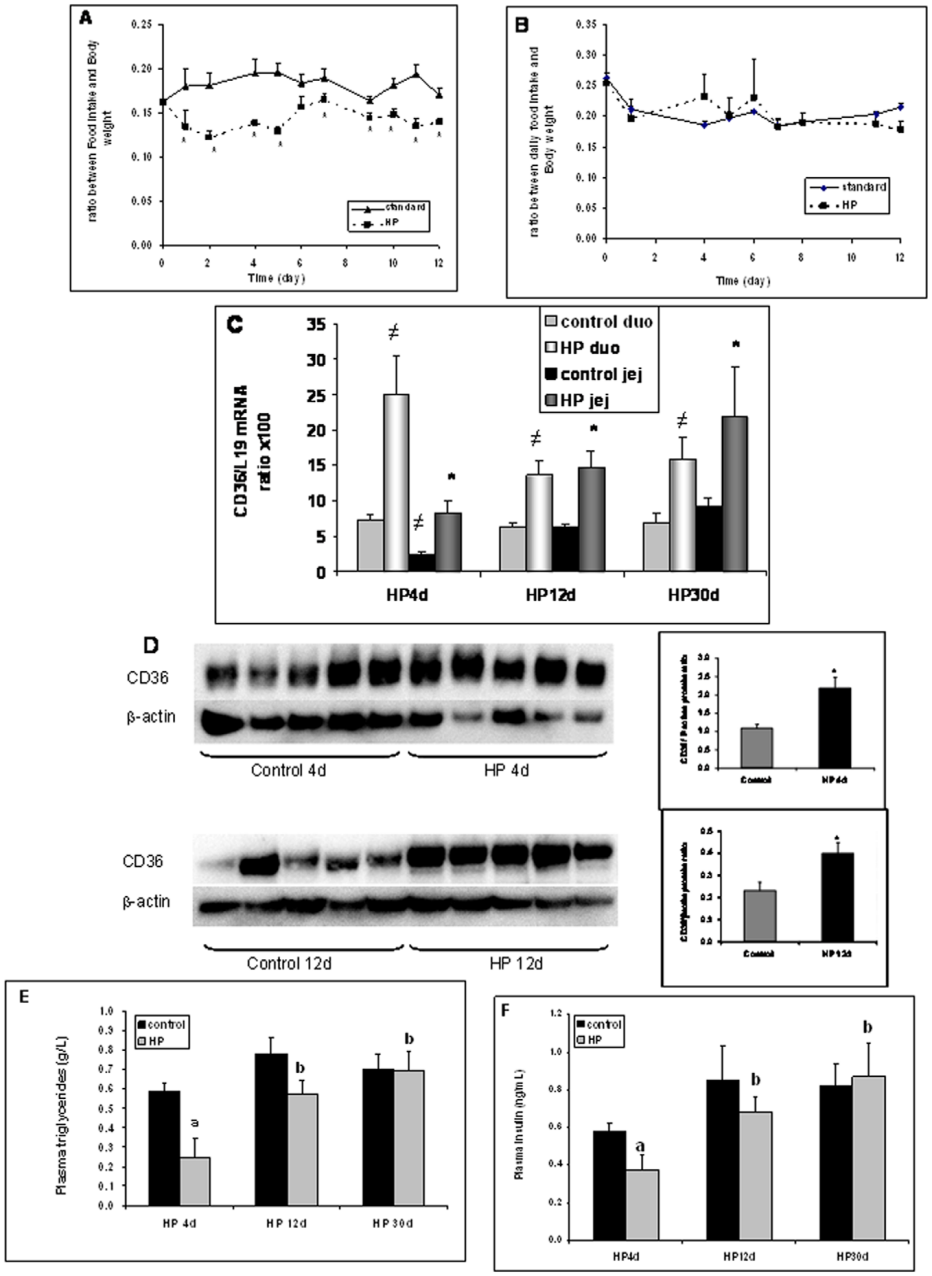
Wild-type and CD36-invalidated mice on Protein-enriched (HP) diet. (**A-B**): Food intake relative to body weight during 12 days of standard or HP diets. (A) corresponds to wild-type (WT) mice and (B) to CD36-invalidated mice (**C**): CD36-encoding mRNA level (relative to L19 gene) in the duodenum and proximal jejunum of WT mice fed standard or HP diet. (≠ P<0.05 relative to duodenum control; * P<0.05 relative to jejunum control; n = 5 to 8). (**D**): Western Blot analyses of proteins prepared from proximal jejunum of WT mice on standard (Control) or HP diet for 4 and 12 days**.** Each lane corresponded to a different mouse. (**E**): Plasma triglycerides measured in control and HPD-fed WT mice. a: P<0.05 differences between control and HPD mice at each period of time; b: P<0.05 relatively to HP 4days. (F): Plasma insulin measured in control and HPD-fed WT mice. a: P<0.05 differences between control and HPD mice at each period of time; b: P<0.05 relatively to HP 4days.

Was the short-term hypophagic effect of an acute lipid load maintained when the mice were adapted chronically to a satietogen diet? To address this question, we studied the effect of the initial administration of an imbalanced diet to mice, on the response to an acute lipid infusion. For this purpose, wild-type mice were subjected for several days to a high-protein diet (HPD). Indeed, it is well known that a chronic HPD is highly satiating and the small intestine is able to adapt its gene expression to its dietary environment [25]. HPD led to a 40% decrease in food intake, from day 1 to day 5. Afterwards, this satiety effect was stabilized at around 15-20% below the control levels (standard chow-fed mice) (Fig 4-A). There were no significant differences in food intake between mice fed either standard chow or HPD in CD36-null mice (Figure 4B). This unexpected data suggests that CD36 gene was required for the satiety effects of HPD. This HP diet up-regulated the intestinal CD36 gene expression (mRNA and protein levels) in proximal intestine of wild-type mice fed on HPD compared to control mice fed on standard laboratory chow (Fig 4-C & D). This effect was more drastic after 4 days of HPD, when food intake was especially low (Fig 4-A), than at days 12 and 30 (Fig 4-C). This effect seems to target specifically CD36, since no induction of the expression of genes encoding other lipid-binding proteins such as L-FABP was found (data not shown). HPD-mediated change in the intestinal CD36 gene expression was associated with a decrease in plasma triglyceride and insulin levels after 4 days of diet, while the decrease in food intake was especially pronounced (Fig 4-E and -F).

**Figure 5:**
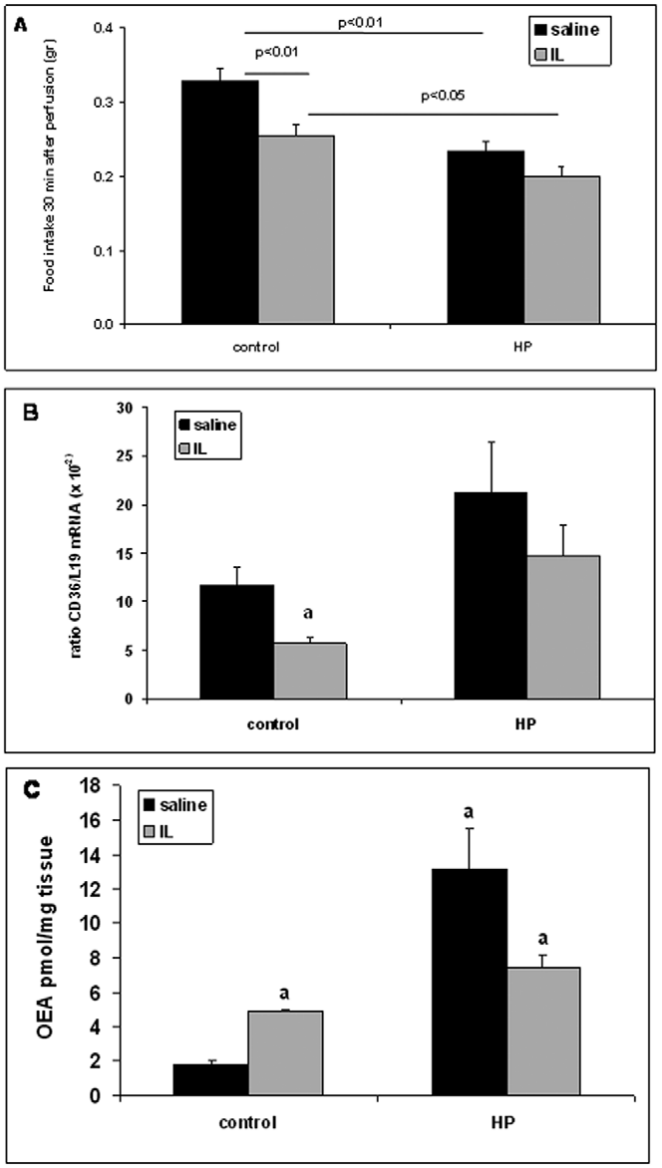
Comparison between WT mice on standard chow (control) and HPD after saline or IL infusion. Mice were fed either standard chow or HP diet 12 days before surgery. Saline and IL infusions were performed after one-week recovery. (**A**): Food intakes were measured 30 min after saline and IL infusions (n = 29 for CT and 17 for HP). (**B/C**): Duodenal CD36 mRNA level (B) and jejunal OEA concentration (C) were measured 45 min after the end of saline and IL infusions. a: P<0.05 relatively to saline infusion in control group.

To explore the impact of this HPD-mediated up-regulation of CD36 gene expression on regulation of food intake, saline or IL infusions into duodenum were performed. As expected, HPD-fed mice had a significant lower food intake levels than control group (Fig 5-A). In contrast to the standard chow-fed mice, in which IL infusion led to a decrease in food intake, no significant satietogen effect of IL was found in mice fed HPD (Figure 5-A). Moreover, the decrease in the duodenal CD36 mRNA levels found in mice fed the control chow and subjected to an IL infusion, was not significantly different in HPD fed animals (Fig 5B).

There was no significant difference in plasma levels of insulin and glucose 45 min after IL infusion and refeeding, compared to saline infusion (Table 3). As well, there was no difference in insulin level between control and HPD-fed mice but a reduction of glycaemia was observed in HPD-fed mice compared to controls (Table 3).

**Table 3:** Plasma insulin and glucose levels in control and HP-fed mice after infusion and refeeding.

	perfusion	insulin (ng/mL)	glucose (mM)
**control**	saline	0.50±0.09	10.8±0.1
	IL	0.58±0.12	10.5±0.3
**HPD**	saline	0.59±0.10	8.5^a^±0.4
	IL	0.51±0.06	8.8^b^±0.5

a: P<0.05 relatively to saline-infused control; b: P<0.05 relatively to IL-infused control.

The lipid messenger OEA was measured in intestinal mucosa 45 min after the end of saline or IL infusions in mice fed the standard chow or HPD. Consistent with previously published data [19], OEA was increased in standard chow-fed mice after IL infusion relatively to saline (Figure 5-C) which is consistent with the IL-mediated decrease in food intake. The concentration of OEA after saline infusions was significantly higher in HPD-fed mice than in standard chow-fed mice, but no additive effect on intestinal OEA levels occurred after IL infusion in contrast with what was found in mice fed the control chow.

## Discussion

Acute infusion of minute amount of lipids (0.12 kCal for 10 min) in duodenum from 5h-fasted mice induced a transient reduction of food intake during refeeding. Our data show that the binding of intestinal CD36 with the pharmacogical drug SSO is sufficient to reproduce this lipid-mediated regulation. Therefore, the ligand binding of intestinal CD36 appears to be a major step in the onset of the satiety triggered by the lipid content of the diet. Along with a decrease in food intake, the lipid infusion induced a decline in CD36 gene expression only at the site of IL infusion (i.e. duodenum). Such a reduction of CD36 mRNA levels has previously been observed after 1hr of intraduodenal infusion of oleate in rats [11]. This long-lasting infusion induced a diminished CD36 expression not only in duodenum but also in the jejunum part of the intestine. Thus, the lipid-induced down-regulation of the CD36 mRNA extended to the two proximal parts of the small intestine after a 1hr-infusion was limited to the duodenum after a shorter infusion. Such differences observed on *cd36* gene regulation could likely be related to the duration of the infusion and the amount of fat available to bind the protein CD36 localized at the site of infusion. In line with a potential role as a lipid receptor, which has been proposed for intestinal CD36 [13], [26], we might therefore draw a parallel between these effects and the well-known down-regulation phenomenon induced by some agonists on their specific receptors [27], [28] that could reflect an activation of the protein CD36. Contrary to this rapid decrease in CD36 gene expression after an acute infusion of lipid, chronic feeding with a high fat diet induced an induction of CD36 mRNA levels [10], [25] suggesting, in this case, an adaptation to the diet to favour lipid absorption.

A satiating effect of dietary fat has previously been described, mainly by using rat infused with a higher amount of fat during longer periods of time [18], [29]. Recently, Schwartz et al [19], using short infusions of a low amount of lipids (0.2 kcal Intralipid during 10 min), have demonstrated a reduction of the consumption of a liquid diet, as measured 30 min after the end of the infusion in wild-type mice, but not in *cd36*-invalidated mice. Our results are in good agreement with these findings. As the transgenic model previously used by Schwartz et al [19] is not a tissue-specific invalidation, we instead preferred to use the sulfosuccinimidyl-oleate (SSO) to limit the inhibition of the CD36 protein to the small intestine. This compound was first described as an inhibitor of LCFA uptake in adipocytes [30]. It specifically binds to the CD36 protein and has been mostly used for *in vitro* studies in several cell models [31], [32] where it alters LCFA transport by CD36 but does not affect the metabolism of these fatty acids [32]. When SSO was infused into the duodenum 5 min prior the saline or IL perfusions, there was no modification of food intake by IL, demonstrating the involvement of the intestinal CD36 in the feeding reduction induced by lipid infusion. However, when SSO was infused prior the saline infusion, a decrease in food intake compared to mice infused Pyrr/saline, was already observed that seems totally dependent on the presence of CD36, as this effect was completely absent in *cd36*-knockout mice. Such a CD36-dependent decrease in food intake induced by SSO alone might depend on its capacity to elicit cell signalling further to binding CD36, in addition to its known role as a competitive inhibitor of fatty acid binding. Such a lipid binding effect has been recently described by Tran et al [13]. Therefore, the reduction in food intake after acute fat load and refeeding was clearly in direct relation with the presence of a functional CD36 in the small intestine.

What is the contribution of this CD36-mediated satietogen effect in a long term satiety-inducing diet? Some studies have demonstrated that the duodenal infusion of lipids in rats inhibits food intake by decreasing meal frequency, but not the meal size [33]. As well, when rats where adapted to a high-protein diet, they reduce the number of meals when compared to rats fed a standard diet [34]. This type of imbalanced diet has been currently used to help humans to lose weight [35] and mechanistic studies have been performed in animal models [36], [37]. Unexpectedly, chronic HP diet, like chronic HF diet [25], induced an increase in CD36 mRNA and protein levels in duodenum and proximal jejunum. These results suggest that the potential capacity to absorb dietary LCFA was increased by this HPD, as well as the capacity of the intestine to signal for a decrease in food intake. Prior to the surgery, all the mice fed on a HPD responded by a decrease in the daily food intake. A reduction in food intake was also observed in HPD mice after saline infusion and refeeding, as compared to infused standard-fed mice, but there was no further decrease after IL infusion. The absence of down-regulation of CD36 mRNA level by IL, in the HPD group might be related to a defective activation of CD36 by lipid infusion and could explain the absence of an additional decrease in food intake.

It has been postulated that OEA acts as a messenger to control the anorexigenic effect of lipids [19]. When directly administered to free-feeding rats, OEA inhibits food intake by delaying meal initiation [38], [39]. In food-deprived animals, OEA delays feeding onset but also reduces meal size [38]. This lipid messenger is produced by proximal small intestine [40] and is an endogenous ligand for peroxisome proliferator-activated receptor-α, PPAR-α [41]. The level of its synthesis is decreased by fasting and increased after refeeding [39]. Moreover, OEA is capable of increasing CD36 mRNA expression in intestinal mucosa [42]. Schwartz et al [19] have previously shown that small-intestine OEA production by mice increases by 30 min after a refeeding which follows a 6h-fast, when compared to the level measured just before refeeding. Herein, we showed that OEA level increased 45 min after IL (*versus* saline) perfusion of mice, as previously demonstrated for IL infused rats [19]. Moreover, when measured 45min after the end of the saline infusion that corresponds to the beginning of refeeding, the jejunal OEA level was much higher in HP group than in control group. We can postulate that this excessive OEA production in response to fasting and refeeding might partly explain the sustained decrease in food intake observed in chronic feeding with HP diet. This higher amount of OEA produced by proximal jejunum in HPD-fed mice *versus* controls (standard chow-fed mice) is in good agreement with the higher expression of CD36 in the proximal small intestine of HPD-fed mice [42]. Moreover, there was no OEA overproduction in response to IL infusion in the HPD group. The reason is probably the excessive jejunal OEA production already induced by refeeding in HPD-fed mice compared to control mice, which might be sub-maximal, making it independent of fat infusion. Indeed, Schwartz et al [19] have demonstrated that OEA is increased after refeeding in standard chow-fed mice, but results described herein indicate that this increase was more pronounced in HPD-fed mice. This effect is CD36-dependent as after *cd36* deletion it was lost [19]. Moreover, the use of CD36 null mice indicated that the hypophagia effect of the high-protein diet was, at least partly, dependent on the presence of CD36. Indeed, other mechanisms have been described to explain the reduction of food intake obtained after HPD [36], [37], [43]. Protein feeding induces a redistribution of endogenous glucose production and, in particular, it increases the amount of glucose released by the small intestine that in turn initiates satiating signals to the brain [36].

We postulate that OEA production may be induced by at least two different mechanisms. An acute intestinal lipid load generates OEA synthesis which requires a functional CD36 protein [19]. In the case of an adaptation to a chronic high-protein diet, wild-type mice exhibit an exaggerated response to refeeding, leading to OEA overproduction. In this case, an acute lipid load will not produce any further increase in OEA production as well as no additional decrease in food intake.

In conclusion, data presented herein, demonstrate that CD36 present in the small intestine is an active player in the lipid-mediated induction of satiety. The experiments using SSO in WT and CD36-null mice, suggest that lipid binding to intestinal CD36 is sufficient to produce a satiety effect. These data suggest that CD36 might control the OEA production/mobilization via an activation of cell signalling [13] which in turn might modulate the activities of NAPE and/or FAAH [19]. Moreover, CD36 could participate in the satiety effect induced by HPD. The small intestine has a high capacity to adapt to different types of diet. This has been demonstrated for lipid-enriched diet, with the acquisition of an increased capacity to absorb fat in response to the enrichment in dietary fat. Herein, we show that intestine can also adapt to a protein-enriched diet partly by CD36-dependent mechanism and participate to the modulation of food intake by several mechanisms including an increase in OEA production and CD36 gene expression. Furthermore, intestine of mice adapted to HP diet have a diminished capacity to modulate their food intake in response to dietary lipids present in the meal. Overall, our study brings new data in favour of the hypotheses advanced by some authors of a specific role of intestinal CD36 as a lipid receptor.
